# Sankey diagrams for macroeconomics: A teaching complement bridging undergraduate and graduate Macro

**DOI:** 10.1016/j.heliyon.2022.e10717

**Published:** 2022-09-23

**Authors:** Gonzalo F. de-Córdoba, Benedetto Molinari

**Affiliations:** a Department of Economics, Universidad de Málaga, Spain; b RCEA (Riverside, CA), USA; c ESCP, France

**Keywords:** Teaching macroeconomics, Sankey diagram, Undergraduate-level macroeconomics, DSGE models, Matplotlib python

## Abstract

There is a widespread call in the academy to teach macroeconomics more homogeneously at the graduate and undergraduate levels. Current state-of-the-art research in macroeconomics obliges teachers of graduate courses to focus on dynamic stochastic general equilibrium (DSGE) models. At the same time, DSGE models have proven too complex for undergraduate-level macroeconomics, which is still grounded on classical model (e.g. IS-LM). In this paper, we propose a tool to bridge the gap between these two conceptual frameworks. Sankey diagrams for macroeconomics provide a coherent graphical analysis representing the aggregate economic activity either defined as in National Accounts, or in classical models, or as an equilibrium outcome of DSGE models. Thus, this tool can be used indifferently in graduate and undergraduate courses providing a guideline for students to recognize the same object of the analysis when jumping from undergraduate to graduate macro.

## 1 Introduction

The dissemination of macroeconomics faces nowadays a urging challenge. There exists a striking gap in academia between the teaching of macro at undergraduate and graduate levels. This gap does not depend on different views or schools or perspectives, but it is rooted in the very own content of today's research in macroeconomics. Cutting-edge research is based on micro-founded, dynamic, sometimes partial but more often general equilibrium models, i.e. Dynamic Stochastic General Equilibrium (DSGE) models, and these models have proven to be too complex to be included among the content of undergraduate macro courses.^1^

As noted by Christiano et al. (2018), DSGE models make up the bulk of current macroeconomic policy, dominate many academic journals, and prevail in most graduate schools where macro courses provide the quantitative tools to cope with these models. One may believe or not that DSGE models are a valid representation of the aggregate economy, as argued by e.g. Blanchard (2016), Krugman (2000), Mankiw (2006). He may even agree with those authors worried about DSGE hijacking the profession while not being valid tools to study macro phenomena (Linde, 2018; Fagiolo and Roventini, 2017). In any case, it seems unrealistic today to imagine a macro graduate course not including DSGE models in the syllabus. As clearly stated by Colander (2018) “Graduate programs today almost exclusively teach DSGE macro”. At the same time, DSGE models constitute a hard challenge for instructors of undergraduate macroeconomics. In the same paper, Colander acknowledges how a gap in macro teaching is created by DSGE models: “the problem is [...] that it is almost impossible to teach DSGE macro at the undergraduate level.” There is a widespread consensus in the profession on this view. According to Ramamurthy and Sedgley "the full-blown micro-founded DSGE framework is out of reach to most intermediate level undergraduate students” (Ramamurthy and Sedgley, 2013). De Araujo et al. stressed the same idea “the degree of technical difficulty involved with these models [DSGE] has prevented them from being widely adopted in undergraduate courses.” (De Araujo, 2013). As a result, “there is almost no connection these days between conventional undergraduate macroeconomics and the macro that is taught in graduate schools.” (Setterfiel, 2018).

All previous authors admit that DSGE models are too complex be included in the syllabus of undergraduate macroeconomics courses. A teacher can push hard on simplifications trying to make DSGE models suitable for undergraduate students, and some effort has been applied in this direction (Solís-García, 2018). The drawback of this approach is that such oversimplification is likely to deliver a blunt tool to tackle macro issues, limiting teachers' ability to explain macro phenomena as observed in actual economies. Undergraduate instructors know well what an asset it is to hinge on students' own experiences to raise interest in a subject. The knowledge of a 20-year-old individual about the macroeconomy and associated macro issues typically comes from news and discussions on TV, newspapers, etc. When it comes to understanding the economic facts behind the news, an oversimplified DSGE model certainty provides fewer insights than classical models like the IS-LM or AD-AS. For this reason, undergraduate macroeconomics is still well grounded in classical models because of their simplicity and intuitiveness. They are efficient tools to introduce new students to the concept of the aggregate economy and their associated empirical facts. The models work well in showing the laws governing empirical regularities in macro data and explaining countrywide macroeconomic issues and challenges. The formalization of classical models is tractable for students with high school level math, basic algebra and arithmetic, a glance at geometry allowing teachers to explain the economics behind aggregate phenomena to students with little mathematical background. In other words, classical models allow students to focus on understanding the economic mechanisms and economy wide relationships among macro aggregates without being stuck in the formalization of micro-founded and dynamic models.

“The macro pedagogy debate” began with these considerations. Not only DSGE models are not included among the content of undergraduate macro courses, but they also differ from classical models in a way so radical that students easily get lost when jumping from undergraduate to graduate macro. Graduate macro does not appear to students as more difficult or a formalized version of undergraduate macro, as it happens with microeconomics or econometrics, but it rather teaches a totally new conceptual framework that at first view has nothing in common with undergraduate macroeconomics. Solving DSGE models requires knowledge of calculus, dynamic programming, and proficiency in computer programming. As argued by Colander (2018), students “don’t know the language [of DSGE models] so they spend all their time learning that, and never really critically deal with the ideas the language is conveying.” Eventually they do not even recognize the basic economic principles, which nonetheless are the same in the two approaches: classical (undergraduate) and marginalist (graduate).

The symposium did not achieve consensus on the best strategy for teaching undergraduate macroeconomics, but all participants agreed on the importance of building bridges between undergraduate and graduate macroeconomics. At least, graduate students should be able to recognize the object of the graduate macro course from their memories of undergraduate macro. Benigno (2018) is an example of this approach. He showed how to extrapolate the basic equations and the graphical analysis of the AS-AD model from a micro-founded New Keynesian DSGE model. This paper goes in the same direction of bridging undergraduate and graduate macro by presenting a graphical analysis of the aggregate economy that can be used as a teaching tool in both undergraduate and graduate macro courses. Specifically, it shows how to represent the aggregate economy by means of a Sankey diagram in which inward flows represent the supply side of the economy (production factors, etc.), and the outward flows represent the demand side (aggregate consumption, aggregate investment, government spending, etc.). In the current practice, the aggregate economy in undergraduate macro courses is typically represented with the circular flow of income diagram, and the graphical analysis associated with classical models depicts aggregate markets in which aggregate demand and supply shift in response to changes in the economic conditions. In graduate macro, the aggregate economy is represented with a set of statistical moments (averages over the considered sample for the long run, second order moments like variance and correlations for the short run), and the associated graphical analysis represents the Impulse Response Functions of macro aggregates characterized by the DSGE model being discussed. So, not only the analytical approaches but also the graphical representations are different.

We believe that having the same graphical tool representing the aggregate economy in both approaches can help students see the correspondences between undergraduate and graduate macroeconomics, and recognize that the object of the analysis is the same. Clearly, Sankey diagrams used in undergraduate macro courses will represent the aggregate economy only from an empirical perspective, providing a quantitative dimension to aggregate variables but failing to explain the causality among these relationships. In the structural approach, Sankey diagrams will instead be responsive to changes in the underlying model parameters, thus showing how macro aggregates depend on agents' behavior (households, firms, government) or on the equilibrium relationship among these variables.^2^ From an instructor’s point of view, the difference between the two approaches will depend on the level of the course. For the basic/intermediate level, where the goal is simply to introduce the students to the visualization of a complex economic system, the empirical approach is adequate. For more advanced courses making use of maximizing agents, the structural approach where the Sankey representation emerges from the resolution of a model may be more adequate, showing how changes in the underlying parameters (technological, preferences, policy) produce a quantitative change in the Sankey representation of the aggregate economy.

## 2 Sankey diagrams for teaching macroeconomics

### 2.1 The Sankey diagram

The Sankey diagram was invented by the Irishman Matthew H. Sankey to represent the functioning of a steam engine. Since then, it has become a standard model used in science and engineering to represent heat balance and energy and material flows. Since the 1990s, the diagram has also been used in life cycle assessments of products (Schmidt, 2008). In general, Sankey diagrams have been used extensively to illustrate the quantitative connections and transformations among different elements in closed systems that receive inputs and return outputs. It gained success and today has its own community featuring implementations in various fields (see www.sankey-diagrams.com). The main characteristic of Sankey diagrams, and most likely the reason for its success, is that diagrams, the width of its inputs and output arrows is proportional to some measure of the output (e.g., energy utilization and energy losses are proportions of energy production). Thus, it provides at the same time a qualitative and quantitative assessment of the relationships represented in the diagram.

Viewed from this angle, an aggregate economy is an object that can be naturally represented with Sankey diagrams. An economy is composed of many different parts that are interconnected in complex ways and devoted to serving a final purpose: the production of goods and services providing welfare for households. Hence, the aggregate economy can be seen as a machine operating with inputs and producing output exactly as a steam engine and, like any engine, it might have leakage (wasted resources), different degrees of efficiency (real or nominal frictions), imbalances (fiscal deficits, unemployment, suboptimal ratio of labor to capital) and, eventually, different magnitudes can be measured in units of output or in terms of welfare. The way different parts are connected defines the underlying model, but the representation of those parts does not change.

In the following, we establish a nomenclature to support coherent representations of the aggregate economy with Sankey diagrams and then we provide three examples of such representations with different levels of complexity. In the simplest case, the diagram simply represents values taken from National Accounts data. In the more complex case, those variables are interrelated by an underlying DSGE model. We hope these visualizations will help students better understand the functioning and the quantitative dimension of the aggregate economy and related macro models.

### 2.2 Establishing the nomenclature

The first step of our proposal establishes a nomenclature to represent macro aggregates using the features of Sankey diagrams in a way that always provides the same meaning. We suggest the following nomenclature:

• **Timeline:** Given that macro aggregates are typically measured with time series, a properly defined Sankey diagram should mimic the dynamic feature. We adopt a standard timeline going from the left to the right in the graph. In particular,
  – **Inward arrows from the left** refer to stock variables (accumulating over time) that are inputs in the productive system. They are *predetermined* in that their value is typically fixed in previous periods by the amount of the corresponding accruing flow variable. For instance, the current nonresidential fixed investment *I*_*t*_, together with the current stock of physical capital *K*_*t*_, determines the stock of tomorrow’s capital *K*_*t*+1_. With this convention, the Aggregate Dynamics of a DSGE model can be represented with a sequence of Sankey diagrams along the timeline.
  – **Outward arrows to the right** refer to the fraction of output that becomes flow variables. In the previous example, investment *I*_*t*_ that accumulates into future capital *K*_*t*+1_.
• **Inward arrows from above** refer to flow variables that are inputs in the productive system, e.g. worked hours *H*_*t*_*.*
• **Outward arrows to the top** represent the finality of the economic system. In an aggregate economy, the most typical example is consumption *C*_*t*_ or public goods *G*_*t*_ that generate utility for the agents.
• **Outward arrows to the bottom:** express frictions and inefficiencies that drain resources from the economy pushing it away from full efficiency. For example, unemployment *U*_*t*_.

### 2.3 Undergraduate macro: Sankey Diagrams with National Accounts

Undergraduate macroeconomics courses like the first year course in an Economics major, or the macro part of Introduction to Economics (Econ 101), typically start with a description of the object of the course: the aggregated economy. These courses follow an empirical approach that focuses on country-wide descriptions of contemporaneous economies and actual macroeconomic issues as in Sloman et al. (2018) and Core (2017). Alternatively, they hinge on more theoretical approaches focusing on general equilibrium and multi-market models as in Blanchard (2017) and Mankiw (2016). In either case, it is very likely that the first description of the aggregate economy that is presented to students is the circular flow of income diagram. The diagram represents a stylized organization of the aggregate economy and constitutes a first example of how to build a model exemplifying a complex reality. It shows which agents exchange goods and money in the economy and the markets where these exchanges take place. Usually, the circular flow of income diagram represents (i) the final goods markets where households and possibly the government are the buyers, and firms are the sellers; (ii) the factor markets, where firms demand production factors (capital and labor) and households supply them, possibly with banks acting as intermediaries.

The circular flow of income diagram provides a clear cut intuition on how the aggregate economy works from a qualitative point of view. Its main drawback is that it does not contain information on the quantities it describes, even though macro students learn that macroeconomic variables have a quantitative dimension. National Accounts are typically presented in the first part of the courses, showing the magnitude of macroeconomic aggregates and the system of National Accounts rules. For this reason, the circular flow of income diagram is usually employed only at the beginning of the course and never revisited at later stages, and it is not employed in more advanced courses like intermediate macro. As a result, the functioning of the aggregate economy and the magnitude of aggregate variables are unrelated concepts in current practice because the circular flow of income diagram is a qualitative tool and thus cannot embed the quantitative dimension of macro variables. For the same reason, the diagram is usually left aside when more formalized models are introduced in the course.

In this section, we present a Sankey diagram designed to close this gap. It represents the aggregate economy and its functioning with a twofold objective. First, it provides a quantitative companion to the circular flow of income diagram, enhancing students' comprehension of the functioning of the aggregate economy. Second, it will provide a bridge to graduate macro, given that in the next sections we show how to use the same graphical analysis to represent the aggregate economy as characterized in DSGE models.

**Figure 1:**
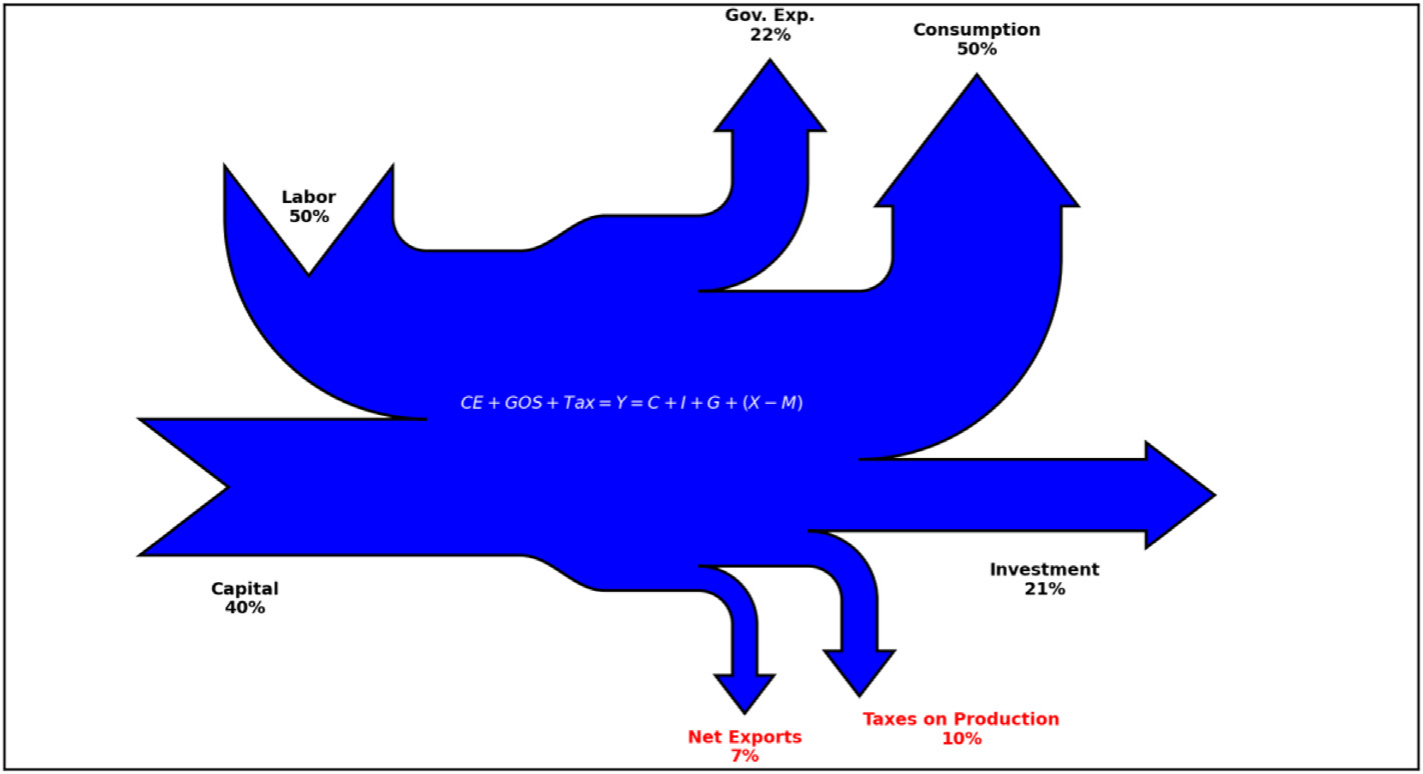
Sankey diagram of the aggregate economy using National Accounts data on GDP.

In representing the aggregate economy with Sankey diagrams, we rely on a few key relationships established in National Accounts data (Table 1). First, we define aggregate output as in National Accounts calculation of *GDP (expenditure approach)*. This definition embeds the equilibrium condition that aggregate demand is equal to aggregate supply, which is then translated to our representation of the economy (reported inside Figure 1). Then, we define aggregate income as in the *GDP (income approach)*. Finally, we equate the two definitions of GDP so that the key condition that aggregate production is equal to aggregate income is also translated to our representation of the aggregate economy.

**Table 1:** National accounts. Holland, 2000–2005.

Description	Sankey D.	Variable	Value
Real GDP (normalized)	**Output**	**Y**	**100%**
*Cons. expenditure of government*	Gov. Exp.	*G*	22.2%
*Private consumption*	Consumption	*C*	49.6%
*Gross private capital formation*	Investment	*I*	21.0%
*External balance of goods and services*	Net Export	*NX*	7.0%
*Compensation of employees*	Labor	*wL*	49.5%
*Gross operating surplus*	Capital	*rK*	40.1%
*Net taxes less subsidies*	Taxes on prod.	*T*	10.4%

Figure 1 depicts a Sankey diagram illustrating the aggregate economy of the Netherlands at the beginning of the XXI century, as described by National Accounts data.^3^ Production factors (Labor and Capital) are represented as inputs (inward arrows) and the component of the aggregate demand as outputs (outward arrows). In Figure 1 they are: Consumption, Investment, and Government expenditure. All variables are expressed in percentages of GDP, and inputs are represented by their shares of aggregate income so that all information uses the same measurement unit. The resulting Sankey diagram represents the aggregate economy as a black box producing goods sold for different purposes and fueled by capital and income. At this stage, the students do not know how the black box works, but they get the idea from the graph that the whole economy operates as an aggregate production function followed by a big market for goods, which are two key elements of the aggregate economy.

According to the interest of the teacher and the availability of time, the Sankey diagram of the aggregate economy can be more granular. On the inflows side, inputs can be as disaggregated as National Accounts data on *GDP (income method)*, for example, by distinguishing between wage and salaries (input) and social security contributions paid by employees (leaking), or the compensation of capital from other voices in Gross Operation Surplus. On the outflows side, National Accounts data on *GDP (expenditures method)* would allow distinguishing, for instance, between households' consumption and general government consumption, or among investments in different sectors.

### 2.4 Graduate macro: Sankey Diagrams with the RBC model

In this section, we provide an example of Sankey Diagrams for graduate macroeconomics by representing the long run equilibrium of a textbook real business cycle (RBC) model. In graduate macro courses, DSGE models are usually introduced using the neoclassical growth model. The model is first taught in its deterministic version (DGE) and then augmented with a stochastic process for technological progress. Eventually, this simple yet fully fledged DSGE model is used to study the Real Business Cycle and thus is commonly known as the *RBC model*.

The equilibrium in DSGE models is characterized by a system of non-linear equations in difference that can be stochastic or deterministic depending on the nature of the model. To study the model economy, two solutions for the system of difference equations are provided: the static and the dynamic solution. The first one characterizes what macroeconomists call the *Steady State* and is used to represent the long run equilibrium of the economy. The second one delivers the *Aggregate Dynamics* and shows how the economy moves from an initial condition to the Steady State, or how it fluctuates around the Steady State in response to a shock. This solution represents the short run behavior of the aggregate economy and is obtained by log-linearizing the stationary and ergodic system of non-linear equations in difference around the Steady State (Taylor approximation of order 1). The approximated model is then solved using one of the many methods available for solving linear equations in difference.

Once the Steady State and Aggregate Dynamics are obtained, the DSGE model is typically tailored to mimic the behavior of a specific economy. One common strategy to accomplish this objective is to calibrate model parameters such that the values of variables in Steady State match the long run averages of their counterparts in macro data. Hence, the same sample economy depicted in Figure 1 can also be represented by means of a DSGE model that matches the National Accounts data reported in Table 1. The possibility of representing the DSGE model using the same graphical tool employed in the previous section establishes a connection between graduate and undergraduate macroeconomics.

It provides, for a student who is told this, a direct comparison of the same object (the aggregate economy) in terms of the same graph but with two different approaches. As compared to the empirical approach used in Section 2.3, the structural approach has the added value of explaining how the black box works, relating macro aggregates to agents' decision rules and mapping empirical facts with deep parameters like preferences and technology. Plus, it helps graduate students to keep a foot on the ground and not get lost in the technicality of DSGE models. Representing the aggregate economy with a Sankey diagram they saw in their undergraduate macro course would remind graduate students that the object of the macroeconomic analysis – micro-founded, reduced form, or empirical – remains the same. In addition, a simple programming code drawing the Sankey representation after computing the equilibrium in DSGE models would show how model parameters changes are related to changes in macro aggregates and thus to the width of the arrows in the Sankey Diagram.

**Figure 2:**
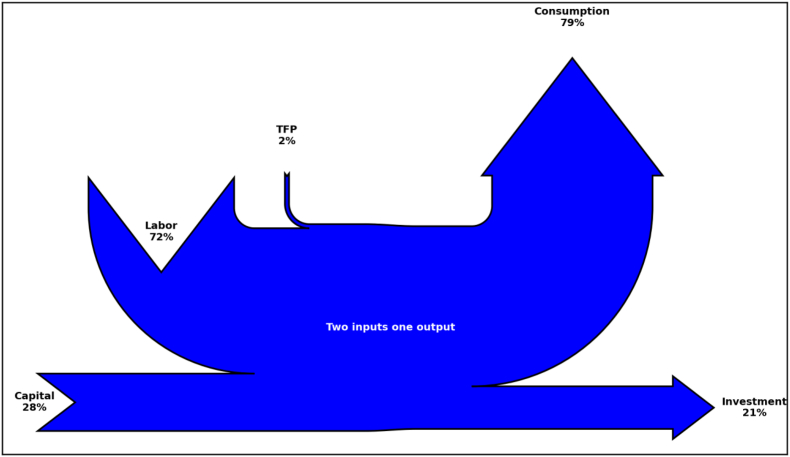
Sankey diagram of the aggregate economy using the textbook RBC model.

**Figure 3:**
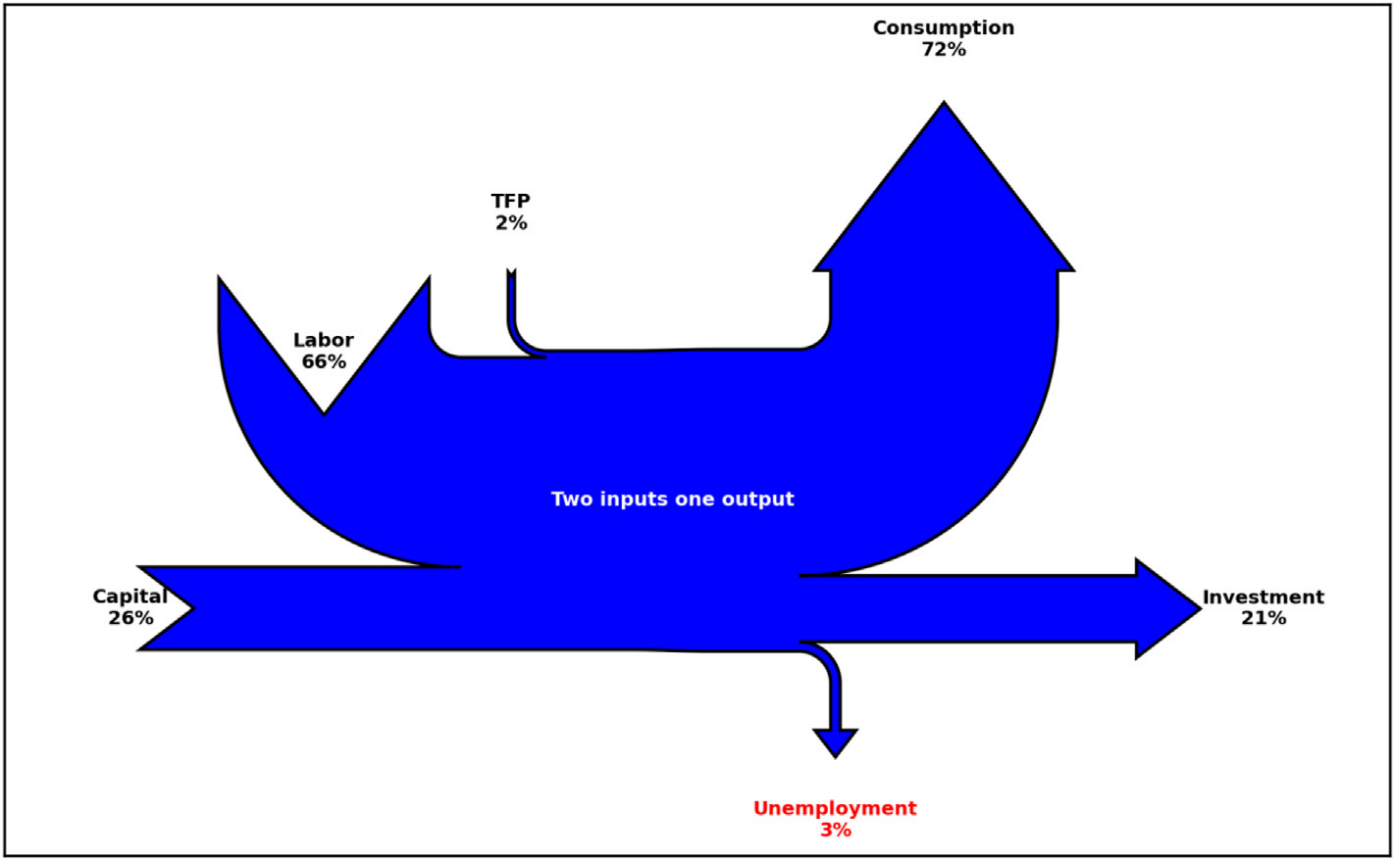
Sankey diagram of the aggregate economy using a neoclassical model with labor market frictions.

In Figures 2 and 3, we illustrate two examples of Sankey diagrams representing the long run equilibrium (Steady State) in two versions of the neoclassical growth model: one without unemployment (RBC model) and the other with unemployment (labor market frictions). Model parameters are calibrated to match a closed economy version of the aggregate economy described in Table 1. Table 2 provides details on the calibration. In both figures, reported values indicate the corresponding variables in Steady State expressed in percentages of output. As in Figure 1, production factors represent Sankey inputs (Capital and Labor shares as computed in Steady State equilibrium) and the uses of production represent Sankey output. In the model, goods sold in the market are used either for consumption or investment purposes.

**Table 2:** RBC model. Parameter calibration.

Description	Parameter	Value	Target
RBC model			
*Discount factor*	*β*	0.96	Subjective discount rate 4*.*1%
*Risk aversion*	*σ*	1	Intertemporal elasticity of substitution 1
*Depreciation rate*	*δ*	0.12	Annual depreciation of capital 12%
*Cobb-Douglas parm.*	*α*	0.28	Labor share of income 72%
*Technology level*	A	2.02	Normalization of GDP (*Y* = 100)
RBC with Labor market frictions		0.1	Matching 10% unemployment
*Unemployment*	u		

Note that only consumption entails welfare gains for households and thus is represented with an upward arrow. Investment, which is the other use of production, accrues in forming tomorrow’s capital input and thus is represented with an horizontal arrow. Following the nomenclature given in Section 2.2, economic inefficiencies are represented as leaking resources in the diagram, as shown by unemployment in Figure 3. Finally, TFP indicates the fraction of total factor productivity needed to obtain a level of aggregate production equal to the aggregate demand given households' endowment and technological restrictions. It is computed endogenously in the model and is treated as a production input itself.

### 2.5 Topics in macro: Sankey Diagrams with medium-size DSGE models

Sankey diagrams provides a good representation of the general equilibrium also in complex models. We illustrate an example with a multisector DSGE model featuring private and public production and economic inefficiencies (de-Códoba et al., 2021). The public sector is represented by a government issuing bonds and levying taxes on goods and income to finance four categories of spending: the public wage bill *θ*^*wl*^, public investment *θ*^*ig*^, public purchases of privately produced goods *θ*^*pg*^, monetary transfers to households *θ*^*zz*^, and public production related expenses *θ*^*gi*^. Public debt *B* can be purchased either by foreign or national investors in a proportion specified by the exogenous parameter Φ. Fiscal income comprises tax and non tax revenues. The model features taxes on consumption *τ*_*c*_, labor *τ*_*l*_ and capital income *τ*_*k*_, social security contributions *τ*_*s*_, and corporate taxes *τ*_Π_. Additional fiscal income is generated from sales of publicly produced goods to households *P*_*gg*_ and from tickets paid by households on in-kind transfers received by the government (1-*S*_*pg*_), where *S*_*pg*_ indicates the fraction subsidized paid by the government.

The private sector is represented by a single firm operating with a Cobb–Douglas technology to produce a homogeneous final good that is purchased for investment or consumption purposes by either households or the government. As in the previous section, we calibrate the model to match the Dutch economy at the beginning of the 2000s. Table 3 reports the extended vector of National Accounts data used to calibrate model parameters, and Table 4 summarizes additional fiscal data needed to calibrate government parameters.^4^

**Table 3:** National accounts. Holland, 2000–2005.

Description	Variable	Value
Normalized (Real) GDP	**Y**	**100%**
*Consumption exp. on public goods of gov’t*	*C*_*gg*_	21.89%
*Consumption exp. on private goods of gov’t*	*G*_*pg*_	7.20%
*Consumption exp. of Households*	*C*_*p*_	49.60%
*Gross private capital formation*	*I*	21.00%
*Compensation of employees*	*wL*	49.50%
*Gross operating surplus*	*rK*	40.10%
*Total government expenditure*	*G*_*tot*_	42.78%
*Total Tax receipts*	*T*_*f*_	35.85%
*Gross General Government Debt*	*B*_*tot*_	60.28%
*Primary government expenditure*	*G*_*prim*_	40.19%
*Implicit interest rate on public bonds*	*rb*	4.00%

**Table 4:** Calibrated policy vector. Holland, 2000–2005.

Parameter	Target	Value
_*τ*_*k*	D2-D211 + D91/B2G B3G: Taxes on production and capital/Gross op. surplus	0.1287
_*τ*_*l*	D51/D11: HHs Income taxes/Wages & Salaries	0.3761
_*τ*_*s*	D12/D11: Social Security Contrib./Wages & Salaries	0.2637
_*τ*_*c*	D211/P31S14S15: Value-added taxes/Final consumption exp.	0.1335
_*τ*_*π*	D51/(B2G-D2D3): Corporations profits taxes/Net op. surplus	0.1493
*θ*^*gi*^	P2: Gov’t Intermediate consumption	0.1512
*θ*^*pg*^	D63: Social transfers in kind	0.1784
*θ*^*ig*^	P5+K2+D9: Gross public capital formation	0.1034
_*θ*_*wl*	D1: Compensation of public employees	0.2248
_*θ*_*zz*	D62 + D3 +D7: Subsidies and monetary transfers	0.3423

**Figure 4:**
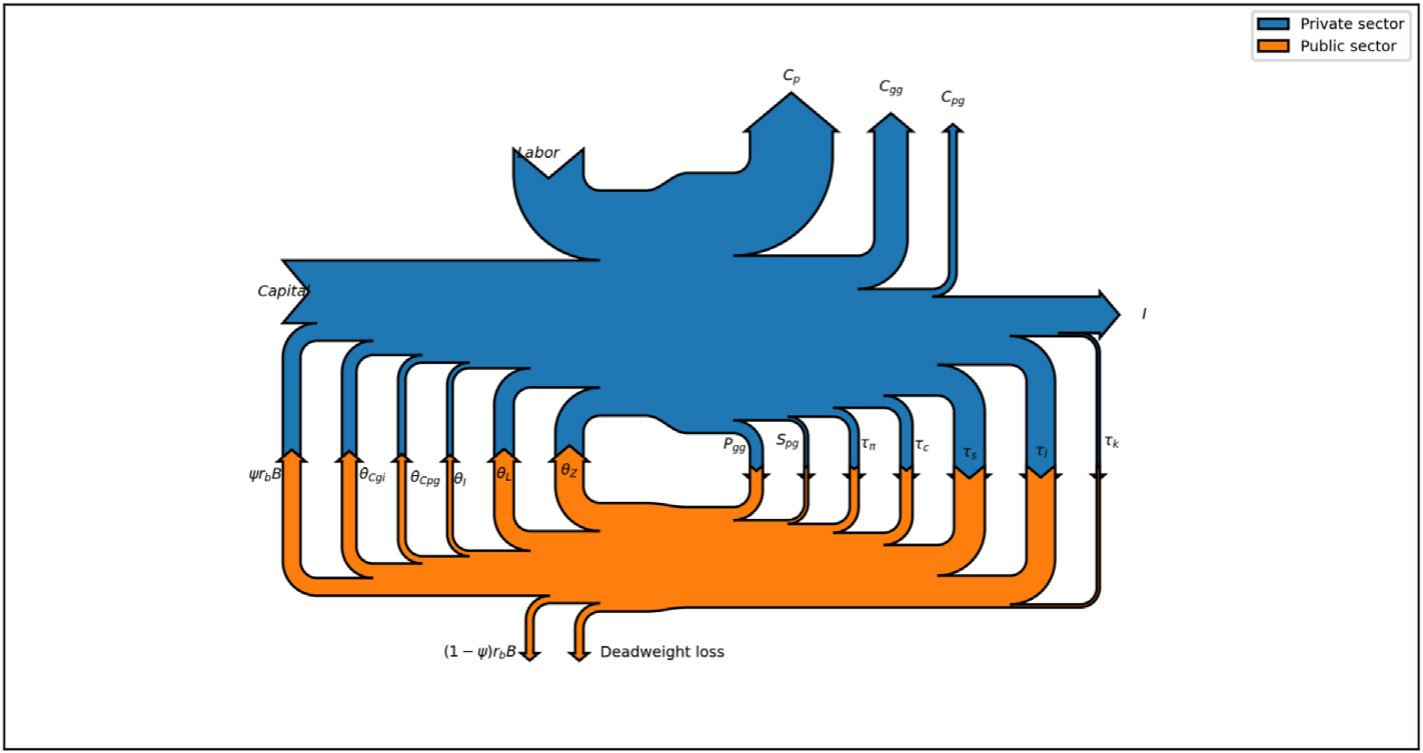
Sankey diagram of the aggregate economy using a two-sector DSGE model.

Figure 4 depicts the Sankey diagram representing the Steady State of this model. Several features are worth mentioning and we shall analyze them in turn. First, by looking at the upward arrows, we can appreciate the three forms of consumption entailing welfare in this model economy. Arrow width shows the relative importance of each welfare source. In Holland, households' own purchases of consumption goods *C*_*p*_ appears twice as big as the sum of households' consumption of public goods *C*_*gg*_ and private goods transferred by the government *C*_*pg*_. Second, investment *I* is represented with an outward arrow to the right because of its nature of flow variable accruing to next period capital, consistent with our nomenclature and previous Sankey diagrams (Figures 1, 2, and 3). Third, inward arrows represent the two production inputs in the model: *Labor* measured as the compensation of employees, which is a flow variable and thus represented with an arrow from above, and *Capital* measured as capital income, which is a stock variable coming from the previous period and thus is represented with an arrow from the left.

Fourth, the government is represented as a stand-alone sector that is interdependent and connected with the private sector through a multiplicity of linkages in both factors and good' markets. These linkages are either withdrawals from the private sector (downward arrows) representing inflows to the government budget constraint or injections to the private sector (upward arrows) representing expenditures for the government.^5^ The width of upward and downward arrows provide a measure of the relative importance of fiscal variables expressed in percentages of GDP. From a visual inspection of the diagram, it is possible to appreciate the size of the government compared to the private sector economy and the relative importance of fiscal revenues and of their uses. Regarding withdrawals, revenues from labor taxation, social security contributions, and VAT taxes appear as first, second, and third most important sources of fiscal income, followed by the revenues from sales of public goods and corporate taxes. Then, the government collects money from ticketing in-kind transfers of privately produced goods to households and capital taxation.

**Figure 5:**
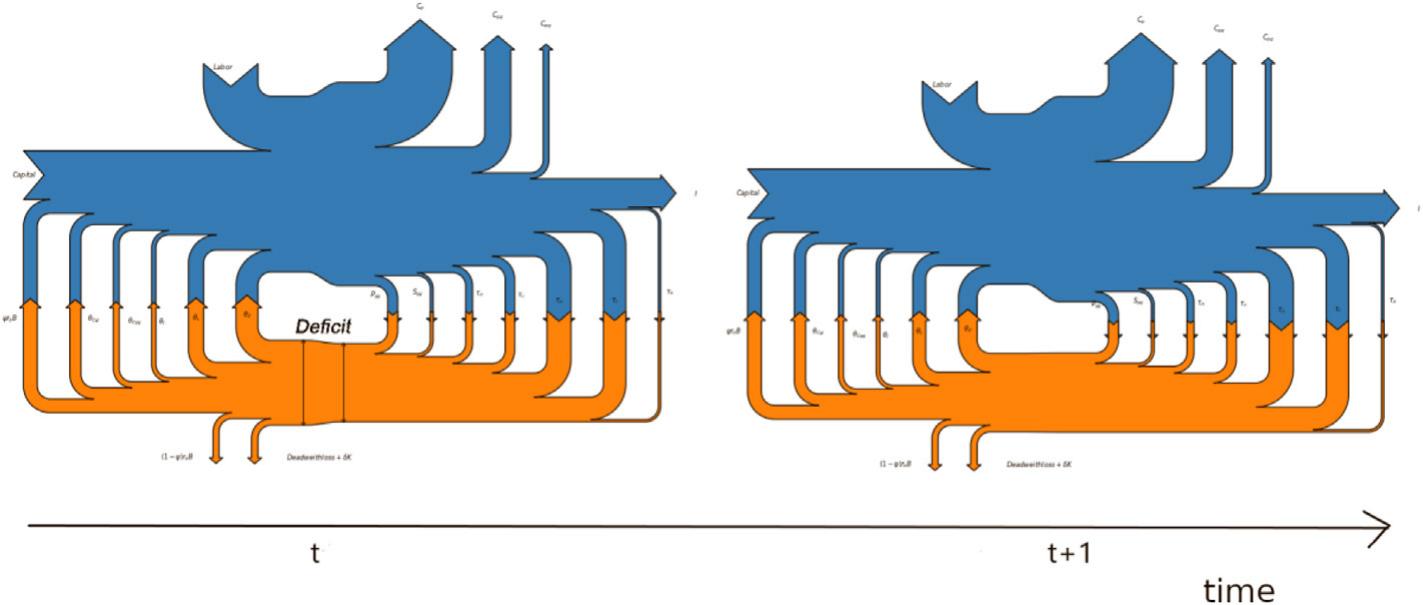
Transition from a fiscal deficit to a steady state.

Fifth, the economic activity of the government comes at a cost. This cost is represented by the loss of economic efficiency (*Deadweight loss*) and interest payments on the fraction of public debt held by nonresident international investors, which is withdrawn from the national economy *((1-Ψ)r*_*b*_*B)*. In accordance with our nomenclature, these leakages are represented with downward exit arrows. As a final remark, note that the economy represented in Figure 4 features macroeconomic imbalances in terms of fiscal deficit, given that the sum of withdrawals (fiscal revenues) is smaller than that of injections (fiscal expenditures). The magnitude of this deficit can be appreciated by the different heights of the two vertical lines depicted in Figure 5. The figure offers an example of how Sankey diagrams can accommodate dynamic adjustments. In period *t*, the aggregate economy features a macroeconomic imbalance that is corrected in *t* +1 by a contractionary fiscal policy reestablishing a balanced budget for the government. The underlying DSGE model quantifies the course of action for the fiscal authority, and the Sankey diagram offers the corresponding visual representation along the timeline.

## 3 Conclusions

We believe that a uniform use of Sankey diagrams as graphical tools representing the aggregate economy can effectively bridge the gap between different levels of macroeconomic teaching. At the undergraduate level, Sankey diagrams can pair the circular flow of income diagram, providing a visualization of the quantitative dimension of macro aggregates as reported in National Accounts. At the graduate level, Sankey diagrams can represent the aggregate economy as defined in DSGE models. A simple programming tool can show how sensitive analysis on model parameters ends up affecting macro aggregates and thus arrow width in the Sankey representation. The level of training does not change the visualization tool, which makes Sankey diagrams useful tools to bridge graduate and undergraduate macroeconomics.

Two final remarks seem appropriate. Sankey diagrams have been around for many decades and have been used for many different purposes. This variety has come with much heterogeneity. Hence, we proposed a nomenclature to provide uniformity, but our proposal is open to improvements introduced by scholars who find this method interesting. Second, to generate the Sankey diagrams presented in this paper we used the *Matplotlib* library of Python. This programming language was chosen for several reasons. Python is free software; it has become a leading programming language; plus, it can be used to compute DSGE models so that the graphical visualization and the underlying theory are seamlessly integrated. In addition, Python codes can be easily embedded in html web pages, flash applications, and Apps used on smartphones and tablets (both *html5* and *Java*). This feature seems most valuable for students practicing and trying out exercises and problem sets.

## Declarations

### Author contribution statement

B. Molinari conceived and designed the experiments; analyzed and interpreted the data; wrote the paper.

G. F. de-Cordoba conceived and designed the experiments; performed the experiments; analyzed and interpreted the data.

### Funding statement

This work was supported by Plan Andaluz de Investigación, Desarrollo e Innovación (PAIDI 2020) grant number P20_01010, and by the European Regional Development Fund (FEDER de Andalucía 2014-2020) grant number UMA18-FEDERJA169.

### Data availability statement

Data included in article/supp. material/referenced in article.

### Declaration of competing interest

The authors declare no conflict of interest.

### Additional information

No additional information is available for this paper.

## Footnotes

1 The gap was acknowledged in an authoritative symposium (“*The macro pedagogy debate”)* hosted in 2018 by the Journal of Economic Education (vol. 49, issue 3).

2 Imagine a set of (*x, y*) pair data represented using a text editor, and the same (*x, y*) set represented using some computing software with the parameters (*a, b*) in the expression *y* = *ax* + *b*. The two representations are equivalent and represent the same line. In the second case, however, the line will be responsive to changes in the parameters (*a, b*).

3 Data are taken from OECD database (https://stats.oecd.org/), section “Annual National Accounts”. Throughout the paper, we use the same data sample to illustrate the Sankey diagrams proposed for different levels of teaching (undergraduate, graduate, topics). We use data on the Dutch economy during the period 2000–2005 because is a small open economy that showed stable fiscal data (see Figures 4 and 5).

4 Data are taken from the OECD database (https://stats.oecd.org/), sections “Annual National Accounts” “Government expenditures by function (COFOG)”, and “Tax database”. Each tax parameter *τ* represents the average tax rate of the corresponding fiscal revenue. Each spending parameter *θ* represents the fraction of public expenditure devoted to the corresponding spending component. Details on data sources, model specification, and the characterization of the equilibrium can be found in de-Có doba et al. (2021).

5 We use here the terminology *withdrawals* and *injections* as in the textbook of Sloman et al. (2018).
