# Supplementary material for: Sankey diagrams for macroeconomics: A teaching complement bridging undergraduate and graduate Macro
Source: Heliyon. 2022 Sep 23;8(9):e10717. doi: 10.1016/j.heliyon.2022.e10717 (PMC9526151; doi:10.1016/j.heliyon.2022.e10717)
Supplement: appendix [file mmc1.pdf]

Companion Appendix to: Sankey Diagrams for Macroeconomics

Gonzalo F. de-Córdoba* Benedetto Molinari†

June 2022

This Appendix provides details on the economic models and the python programs used to produce the Sankey Diagrams presented in the main text. Each section of the Appendix refers to one of the figures presented in the companion paper that are indicated maintaining the same numbering. For each figure, we provide the underlying economic model and we discuss the most important aspects of the python coding. We also include among the supplementary material a compressed folder with all the routines used to produce the figures in the paper.

1 Figure 1

1.1 Model

The first Sankey Diagram presented in the paper (Section 2.3) is built using no underlying model and only exploiting data from National Accounts (reported in Table 1 of the main text). As explained in the paper, this is the simplest use of a Sankey Diagram to represent an aggregate economy and the python routine only takes data as input and produces the Sankey Diagram as output.

1.2 Code

The script generating Figure 1 is stored as "Figure1.py". It can be executed from any python installation (v. 3) using the following command in the prompt:

*Department of Economics at the Universidad de Málaga, and ESCP. Email: gfdc@uma.es
†Department of Economics, Universidad de Málaga and RCEA. Email: bmolinari@uma.es

```
>>exec(open('Figure1.py').read())
```

or from a GNU/Linux terminal as $ Figure1.py.[1] The file "Figure1.py" opens with an attribution and a copyright notice that is the same in all of the other routine used for this paper. It reads as follows:

```
"""
NAME
      Figure1.py takes data from Table 1 in the paper and represents
      an economy using the Sankey diagram as explained in the paper
SYNOPSIS
      Execute at the python prompt as >> exec(open("Figure1.py").read()),
      or python Figure1.py at the GNU/Linux terminal
DESCRIPTION
      Has no dependencies
AUTHOR
      Gonzalo F. de Cordoba and Benedetto Molinari
LICENSE
      Copyright © 2018 License  GPLv3+:  GNU GPL version 3 or later
      <http://gnu.org/licenses/gpl.html>.
      This  is  free  software:  you  are free to change and redistribute it
      There is NO WARRANTY, to the extent permitted by law.
"""
```

In the following section, the routine imports two libraries from **Matplotlib**. This last is a plotting library for creating static, animated, and interactive visualizations in Python. Matplotlib can be used in Python scripts, the Python and IPython shell, web application servers, and various graphical user interface toolkit like Tkinter and awxPython. The first library is **Pyplot**, which is a Matplotlib-specific module providing a MATLAB-like interface [2]. The second library is **Sankey**, which is the module to produce Sankey Diagrams [5]. The code reads as follows,

```
import matplotlib.pyplot as plt
from matplotlib.sankey import Sankey
```

Next, the routine code requires the user to input data to calibrate the diagram. In the case of an aggregate economy, these values can be taken from National Accounts as discussed in the companion paper. We used the following values for macro aggregates

```
# Calibration
y = 100
wl = 49.5
rk = 40.1
G = 22.2
```

[1] All Python files presented in this appendix can be opened using any of these two methods.

```
c = 49.6
i = 21
Nx = y-c-i-G
T = 10.4
```

Next, it follows the commands specific to the Sankey library, i.e.

```
fig0 = plt.figure()
ax0 = fig0.add_subplot(1, 1, 1, xticks=[], yticks=[], title="  ")
sankey = Sankey(ax=ax0, scale=0.01, offset=0.2, head_angle=75,
                format='%.0f', unit='%', shoulder=0.05)
print(sankey.tolerance)

sankey.add(flows=[-G, rk, wl, -c, -i, -T, -Nx],
           labels=['Gov. Exp.', 'Capital', 'Labor', 'Consumption',
                   'Investment', 'Taxes on Production', 'Net Exports'],
           orientations=[1, 0, 1, 1, 0, -1, -1],
           pathlengths=[0.25, 0.25, 0.25, 0.25, 0.25, 0.25, 0.25],
           patchlabel=r'                      $wL+rK+T = Y = C+I+G+(X-M)$',
           facecolor='blue')  # Arguments to matplotlib.patches.PathPatch()
diagrams = sankey.finish()
print(diagrams[0].patch)
```

The command **plt.figure()** is the Matlab-like command to create a figure object. Then we **add** a 1×1 matrix of subplots, that is, one canvas to the figure with a set of properties for the axis. Next, the constructor of the class **Sankey** is called. With the method **add** we add the arrows that take values $\{-G, rk, wl, -c, -i, -T, -Nx\}$ with the corresponding labels. The sign of the variable tells the module if the arrow exits (-) or the arrow enters (+). The orientations take the value 0, for a flat arrow, 1 for an upwards arrow and -1 for a downwards arrow. So, for example, a flow of magnitude $-G$ goes outside pointing up. The numerical value, tells the Sankey module the width of the arrow.

Finally, the elements of the Sankey plot are created with the command **sankey.finish()**. These objects are called **diagrams** in this script. This elements that we have called diagrams have a set of properties like the color of the text, and the weight of the letter type and also the position in the diagram. The code ends with the command **show()** from the module **pyplot**, and it orders the computer to render the graph if a pop-up window

```
diagrams[0].texts[6].set_color('r')
diagrams[0].texts[6].set_fontweight('bold')
diagrams[0].texts[6].set_fontsize(6)
diagrams[0].texts[6].set_position([diagrams[0].texts[6].get_position()[0],
                                   diagrams[0].texts[6].get_position()[1]+0.1])
plt.show()
```

2 Figure 2

2.1 Model

Figures 2 and 3 rely on the same underlying mode, which is the neoclassical growth model with perfect competition and real prices. This model is typically used as prototype DSGE model in textbooks and courses of graduate macro (see e.g. [3]). Its long-run equilibrium is described by the (non-stochastic) static solution of the system of equations in difference characterizing the equilibrium in the aggregate economy. This static solution, called the Steady State of the economy, is thus represented by the following equations:

$$
\begin{aligned}
1 &= \beta\left(1-\delta+\overline{R}\right) && (1)\\
u'(\overline{L}) &= \overline{W}u'(\overline{C}) && (2)\\
\overline{C}+\overline{I} &= \overline{R}\cdot\overline{K}+\overline{W}\cdot\overline{L} && (3)\\
\overline{K} &= (1-\delta)\overline{K}+\overline{I} && (4)\\
\overline{R} &= (1-\alpha)\overline{A}\cdot\overline{K}^{-\alpha}\overline{L}^{\alpha} && (5)\\
\overline{W} &= \alpha\overline{A}\cdot\overline{K}^{1-\alpha}\overline{L}^{\alpha-1} && (6)\\
\overline{Y} &= \overline{A}\cdot\overline{K}^{\alpha}\overline{L}^{1-\alpha} && (7)
\end{aligned}
$$

which can be solved either analytically or numerically by the mean of a fixed-point algorithm. In the case of DSGE models, the routine needs two inputs from the user. First, data in the form of calibration targets, which are used to assign numerical values to model parameters. Second, the system of steady state equations. In the case of the textbook DSGE model used for Figures 2 and 3, the steady state admits an analytical solution, which is pre-written in the code. In the case of larger and/or more complex models, the steady state has to be computed using numerical methods. Thus, the routine embeds a Newton-Rapson algorithm to compute numerically the steady state using the system of equations introduced by the user. Eventually, the program returns the Sankey Diagram representing the equilibrium relationships between macroeconomic aggregates in the long run as output.

2.2 Code

Routine “Figure2.py” opens with the same comments as “Figure1.py”, followed by the same import commands for Matplotlib libraries. This section is followed by the programming code for the calibration, in which the user introduces the calibration targets. In our case,

```
# Calibration
yss = 100  # A*kss**alpha  ``Normalization''
iss = 21  # delta*k = 21   ``Steady State investment''
lss = 30  #                ``Total labor''
delta = 0.12 #             ``Depreciation rate''
beta = 0.96 #              ``Discount factor''
rss = 1/beta-(1-delta) #   ``Implied equilibrium rental rate''
kss = iss/delta #          ``Implied capital stock''
```

```
alpha = rss*kss/yss #      ''Implied capital share''
A = yss/(kss**alpha*lss**(1-alpha)) # ''Implied TFP''
```

As next step, the equations characterizing the equilibrium are used to compute the macro aggregates generated in the model like aggregate consumption, aggregate output (GDP), etc.

```
# Model equations
urate = 0.0
lssu = (1-urate)*lss
yss = A*kss**alpha*lssu**(1-alpha)
rss = A*alpha*kss**(alpha-1)*lssu**(1-alpha)
wss = A*(1-alpha)*kss**alpha*lssu**(-alpha)
css = yss-delta*kss
```

Once the model is solved, the routine embeds the lines of code to generate the Sankey Diagram. They are the same as in "Figure1.py", as described in previous section. The macroeconomic aggregates depicted in Figure 2 are defined as follows:

- $\frac{css}{yss}$ is "Consumption"
- $\frac{A}{yss}$ is "TFP"
- $\frac{wss \cdot lssu}{yss}$ is "Labor" (share of income)
- $\frac{rss \cdot kss}{yss}$ is "Capital" (share of income)

3 Figure 3

Figure 3 stems from the same routine "Figure2.py", but setting the variable `urate` $\in [0, 1)$ to a value different from zero. It represents the same model economy as shown in Figure 2 but in the presence of unemployment, which is used as an example of wasting-resource leaks from an economic system. The red leak arrow in this Sankey Diagram is computed as $\frac{urate \cdot lss}{yss}$ and it is indicated as "Unemployment".

4 Figure 4

4.1 Model

The economy depicted in Figure 4 represents the long run equilibrium in a small open economy DSGE model described in [1]. To make this appendix more readable, we only number the equations from the model that are referred to in the script of the program generating Figure 4. The full model and all modeling details are available in [1]. Coding notation follows the one of the equations presented here and, for the sake of clarity, we also added a map inside the python routine to recall the correspondences.

The model is focused on the effects of the government in the aggregate economy and provides an example of how to represent a two-sector economy with Sankey Diagrams. To develop the public sectors it starts using the typical government's budget constraint used in DSGE models

$$G_{p,t} + (1 + R_t^B)B_t = T_t + B_{t+1}$$

which states that government spending plus the stock of existing debt needs to be financed either with debt B_{t+1} or with fiscal revenues T_t. Government's budget constraint can be written in a more compact form using the definition of total government spending $G_t = G_{p,t} + R_t^B B_t$. Then,

$$G_t - T_t = \Delta B_{t+1} \tag{8}$$

Next, it details both government expenditures and tax revenues. Total fiscal revenues are

$$\begin{aligned} T_t &= \tau_t^c C_{pp,t} + (\tau_t^l + \tau_t^s)(w_{p,t}L_{p,t} + w_{g,t}L_{g,t}) + \\ &\quad + \tau_t^k (R_t - \delta_p)K_{p,t} + \tau_t^\pi \Pi_t + (1 - s_{p,t})C_{pg,t} + (p_{g,t} - s_{g,t})C_{gg,t} \end{aligned} \tag{9}$$

where $C_{pp,t}$ and $C_{pg,t}$ indicate households' consumption of privately produced goods, respectively, purchased by households or purchased by the government and provided to households. Note that government gets revenues from the provision of both private and public goods. In particular, for the private good the government pays the whole amount of $C_{pg,t}$ (see equation 4.1) and then collects the unsubsidized fraction of the price $(1 - s_{p,t})$. For the public good, the government directly sells it at the price $(p_{g,t} - s_{g,t})$ that already includes the subsidy $s_{g,t}$. Finally, private prices are represented by wages $w_{p,t}$ and rental rate of private capital r_t, whereas $L_{p,t}$ and $K_{p,t}$ represent, respectively, private labor and capital. Π_t are private firms' profits. Taxes are τ_t^c for the VAT tax, τ_t^l and τ_t^s are labor tax and social security tax respectively, whereas τ_t^k stands for capital tax and τ_t^π are corporate taxes.

Government expenditures are divided in five chapters of spending using the following definitions

$$C_{gi,t} = \theta_1 G_{p,t} \quad \text{Final gov. intermediate cons.} \tag{10}$$

$$C_{pg,t} = \theta_2 G_{p,t} \quad \text{Final gov. consumption} \tag{11}$$

$$I_{g,t} = \theta_3 G_{p,t} \quad \text{Gov. investment} \tag{12}$$

$$(1 + \tau_t^{ss})W_{g,t}L_{g,t} = \theta_4 G_{p,t} \quad \text{Gov. labor payments} \tag{13}$$

$$Z_t = \theta_5 G_{p,t} \quad \text{Gov. transfers} \tag{14}$$

with $\theta_1 + \theta_2 + \theta_3 + \theta_4 + \theta_5 = 1$. In other words, we assume that public spending on goods, services and monetary transfers to households are constant proportions of total spending, and these proportions are constant all along the exercise, so that government's income and expenditure are fully parametric.

Private output $Y_{p,t}$ is thus produced using a technology employing private capital $K_{p,t}$, public capital $K_{gp,t}$, private labor $L_{p,t}$ and public labor $L_{gp,t}$

$$Y_{p,t} = A_{p,t}F(K_{p,t}, K_{gp,t}, L_{p,t}, L_{gp,t})$$

For running the python programs we have made use of the following technology

$$Y_{p,t} = A_{p,t} K_{p,t}^{\alpha_p} K_{gp,t}^{\alpha_g} \left(\mu L_{p,t}^{\eta} + (1-\mu) L_{gp,t}^{\eta}\right)^{\frac{\alpha_l}{\eta}} \tag{15}$$

Where private and public capital laws of accumulation are described by the following permanent inventory equations:

$$K_{p,t+1} = (1-\delta_p) K_{p,t} + I_{p,t} \tag{16}$$

and total public capital $K_{g,t}$ is

$$K_{g,t+1} = (1-\delta_g) K_{g,t} + I_{g,t} \tag{17}$$

We include public production $Y_{g,t}$ in the model using a Cobb-Douglas production function, i.e.

$$Y_{g,t} = \Gamma C_{gi,t}^{\gamma} L_{gg,t}^{(1-\gamma)} \tag{18}$$

In equilibrium, public output $Y_{g,t}$ is entirely given to households in the form of consumption and, accordingly, we define

$$C_{gg,t} = p_{g,t} Y_{g,t} \tag{19}$$

The zero profit condition of the public sector, and the accounting rule that establishes that the goods produced by the government are always valued at factor costs imply the following solution:

$$p_{g,t} Y_{g,t} = C_{gi,t} + (1+\tau_t^s) w_{g,t} L_{gg,t} \tag{20}$$

where $p_{g,t}$ is the relative price of public goods in terms of private goods.

We assume that privately produced goods are sold in perfectly competitive markets and private production technology has constant returns to scale. Then, firms maximizing profits problem

$$\underset{\{K_{p,t},L_{p,t}\}_{t=1}^{\infty}}{Max} \Pi_t = Y_{p,t} - (1+\tau_t^s) w_{p,t} L_{p,t} - r_t K_{p,t}, \tag{21}$$

delivers the following inverse demand functions for private factors

$$r_t = F_{K_p} \tag{22}$$

$$(1+\tau_t^s) w_{p,t} = F_{L_p} \tag{23}$$

Using previous definitions and notation, government spending in the model is

$$G_t = (1+\tau_t^s) w_{g,t} L_{g,t} + I_{g,t} + Z_t + C_{pg,t} + C_{gi,t} + R_t^B B_t$$

Finally, we impose the usual market clearing conditions on the labor market,

$$L_t = L_{p,t} + L_{g,t} \tag{24}$$

and

$$L_{g,t} = L_{gp,t} + L_{gg,t} \tag{25}$$

The government is assumed to use a rule that keeps a constant proportion of public workers working in the production of public goods and public workers working in the general administration.

$$L_{gp,t} = \theta_{lgp} L_{g,t} \tag{26}$$

The rest of the world for this economy is modeled as a single international banker whose objective is to maximize the discounted dividend x_t obtained from the asset holdings of government bonds. The discount factor is β, identical to the consumer's discounting parameter. Purchases of government bonds are denoted by b_t. Of course, supply and demand are equal at all times, so $B_t = b_t$.

$$\max_{x_t} \sum_{t=0}^{\infty} \beta^t x_t$$
$$s.t.\ b_{t+1} - b_t + x_t = w^I + r^b b_t$$

Where w^I is a constant endowment.

From the above problem we obtain

$$\beta(1 + r^b) = 1$$

From the above equation and (22) we obtain a non arbitrage steady state condition

$$(1 - \tau^k)(r_t - \delta_{K_p}) = r^b \tag{27}$$

The net real return to capital has to equate the real return of the government bond, including any risk premium.

The set of equations describe a complete general equilibrium that is represented for the steady state of the economy with a Sankey Diagram. The total size of the public sector is a calibrated parameter that follows from

$$G_t/Yt = ratGY \tag{28}$$

Walras's Law is satisfied at all times[2], and therefore the above set of equations determine that:

$$Y_t + B_{t+1} - (1 + r^b)B_t = I_{p,t} + I_{g,t} + C_{gi,t} + C_{pg,t} + C_{p,t} \tag{29}$$

These are the 22 equations that appear on the code below. As a final remark, we allow in the Sankey representation of the economy for a certain fraction of debt payments to flow out of the economy as a total loss for the system

4.2 Code

The model developed in this section requires the implementation of the Newton-Raphson algorithm. The method **fsolve** from the library scipy is used and therefore needs to be imported. The following snippets of code correspond to **Figure4.py**

[2]See the references for a proof in [1].

```
import numpy as np
from scipy.optimize import fsolve
import timeit
from Figure4cpo import Figure4cpo
from tabulate import tabulate
import matplotlib.pyplot as plt
from matplotlib.sankey import Sankey
```

The library NumPy (Numerical Python) is an open source Python library that's used in almost every field of science and engineering. It's the universal standard for working with numerical data in Python, and it's at the core of the scientific Python and PyData ecosystems [4].

After setting the values of observed macroeconomic variables, and after normalization (for example, Steady State GDP in the model economy is set to $Y_{ss} = 100$), we obtain a vector of parameters that is passed to the solver. The computational time shows in the screen.

```
# Compute a steady state with those parameters
tic = timeit.default_timer()
x0 = [Kp, Kg, Lp, Lg, B]
ratGY = G/Y
param = [alphap, alphag, alphal, deltap, deltag, thetaCgi, thetaL,
         thetaCpg, thetaI, thetaLgp, mu, eta, tauc, taul, tauk, taus,
         taupi, sgg, spg, L, A, ratGY, rb, gamma, Gamma]

def g(x):
    return Figure4cpo(x, param)

sol = fsolve(g, x0,  xtol=crit)
toc = timeit.default_timer()
print("time=%f" % (toc - tic))
```

Once the Newton-Raphson exists, four variables (the state variables) are determined, and the rest of the model is also determined.

```
# Variable's binding
Kpss, Kgss, Lpss, Lgss, Bss = (sol[i] for i in range(5))

# Ecuations of the model
Lgpss = thetaLgp*Lgss                 # Public labor producing GDP
Lggss = Lgss-Lgpss                    # Public labor producing public goods
Ipss = deltap*Kpss                    # Private investment
Igss = deltag*Kgss                    # Public investment
Lag = mu*Lpss**eta+(1-mu)*Lgpss**eta # Labor component in GDP
Kag = Kpss**alphap*Kgss**alphag      # Capital component in GDP
Yss = A*Kag*Lag**(alphal/eta)        # Total GDP
```

```
Gss = ratGY*Yss                       # Total government expenditures
rss = alphap*A*Kpss**(alphap-1)       # Rental rate of capital
    *Kgss**alphag*Lag**(alphal/eta)
wss = (alphal*A*mu*Lpss**(eta-1)      # Wages
    *Kag*Lag**(alphal/eta-1))/(1+taus)
Cgiss = thetaCgi*Gss                  # Gov. exp. into investment
Ygss = Gamma*Cgiss**gamma             # Public output
     *Lggss**(1-gamma)
Cggss = Ygss                          # Private consumption of public goods
pggss = (Cgiss+(1+taus)*wss*Lggss)    # Price of the public good w.r.t. private
        /Ygss
Zss = thetaZ*Gss                      # Gov. exp. in transfers to consumers
Cpgss = thetaCpg*Gss                  # Gov. exp. in public wages
Cpss = Yss-rb*Bss-Ipss-Igss           # Private consumption
       -Cgiss-Cpgss
PIss = Yss-(1+taus)*wss*Lpss-rss*Kpss# Firm's profits
IFss = tauc*Cpss+(taul+taus)*wss*L    # Fiscal revenues
     +tauk*(rss-deltap)*Kpss +
     taupi*PIss+(1-spg)*Cpgss+(pggss-sgg)*Cggss
```

Right after computing the solution of the model, the program tabulates the output to the screen in a nice table

```
# Print tables with the steady state
table1 = [["Tech1", "alphap", alphap, "alphag", alphag, "alphal", alphal],
          ["Tech2", "Gamma", Gamma, "gamma", gamma, "eta", eta, "mu", mu],
          ["Tech3", "A", A, "deltap", deltap, "deltag", deltag], [],
          ["Tax1", "tauc", tauc, "tauk", tauk, "taul", taul],
          ["Tax2", "taupi", taupi, "taus", taus], [],
          ["Theta1", "thetaCgi", thetaCgi, "thetaL", thetaL, "thetaCpg",
          thetaCpg], ["Theta2", "thetaZ", thetaZ, "thetaI", thetaI,
                      "thetaLgp", thetaLgp], [], ["Prices", "sgg", sgg,
                                                  "spg", spg], [],
          ["Other", "beta", beta, "rb", rb, "L", L]]

print(tabulate(table1))

table2 = [["Output1", "Cp", Cpss, "Cpg", Cpgss, "Cgg", Cggss],
          ["Output2", "Y", Yss, "Cgi", Cgiss, "Yg", Ygss], [],
          ["Input1", "Kp", Kpss, "Kg", Kgss, "Ip", Ipss, "Ig", Igss],
          ["Input2", "Lp", Lpss, "Lg", Lgss, "Lgp", Lgpss, "Lgg", Lggss],
          [], ["Prices", "r", rss, "w", wss, "pgg", pggss], [],
          ["Balance", "G", Gss, "IF", IFss, "B", Bss, "rb*B", rb*Bss]]
print(tabulate(table2))
```

In order to be able to keep track of the inflows and outflows of such a large model, we number each of the items in the following assignments:

```
in1, out1 = psi*rb*Bss/Yss, -psi*rb*Bss/Yss
in2, out2 = thetaCgi*G/Yss, -thetaCgi*G/Yss
in3, out3 = thetaCpg*G/Yss, -thetaCpg*G/Yss
in4, out4 = thetaI*G/Yss, -thetaI*G/Yss
in5, out5 = thetaL*G/Yss, -thetaL*G/Yss
in6, out6 = thetaZ*G/Yss, -thetaZ*G/Yss
in7, out7 = tauk*(rss-deltap)*Kpss/Yss, -tauk*(rss-deltap)*Kpss/Yss
in8, out8 = taul*wss*L/Yss, -taul*wss*L/Yss
in9, out9 = taus*wss*L/Yss, -taus*wss*L/Yss
in10, out10 = tauc*Cpss/Yss, -tauc*Cpss/Yss
in11, out11 = taupi*PIss/Yss, -taupi*PIss/Yss
in12, out12 = (1-spg)*Cpgss/Yss, -(1-spg)*Cpgss/Yss
in13, out13 = (pggss-sgg)*Cggss/Yss, -(pggss-sgg)*Cggss/Yss
in14, out14 = wss*L/Yss, -Cpss/Yss
in15, out15 = rss*((1-deltap)*Kpss+(1-deltag)*Kgss)/Yss, -sgg*Cggss/Yss
out16 = -spg*Cpgss/Yss
out17 = 0.0005*Yss
out18 = (1-psi)*rb*Bss/Yss
out19 = -(Igss+Ipss)/Yss
```

And we are now ready to invoke the Sankey library in the following commands:

```
fig1 = plt.figure()
ax = fig1.add_subplot(1, 1, 1, xticks=[], yticks=[], title="  ")
sankey = Sankey(ax=ax, unit=None)
```

Where we first create a matplotlib figure called fig1. Then we create an axis object and **add** it to the axis object. Finally, we call the sankey diagram constructor **Sankey** creating an instance called sankey.

Then we use the flows and the labels to each arrow with the following commands:

```
flows1 = [in1, in2, in3, in4, in5, in6, out7, out8, out9, out10,
          out11, out12, out13,  in14, in15, out14,
          out15, out16, out19]
labelsTau = [r'$\psi r_{b}B$', r'$\theta_{Cgi}$', r'$\theta_{Cpg}$',
             r'$\theta_{I}$', r'$\theta_{L}$', r'$\theta_{Z}$', r'',
             r'', r'', r'', r'', r'', r'', r'$Labor$', r'$Capital$',
             r'$C_{p}$', r'$C_{gg}$', r'$C_{pg}$', r'$I$']
sankey.add(flows=flows1, label='Private sector',
           orientations=[-1, -1, -1, -1, -1, -1, -1, -1, -1, -1, -1, -1,
                         -1, 1, 0, 1, 1, 1, 0],
           labels=labelsTau)
flows2 = [out1, out2, out3, out4, out5, out6, in7, in8, in9, in10, in11,
          in12, in13, -out17, -out18]
labelsCita = [r'', r'', r'', r'', r'', r'', r'$\tau_{k}$', r'$\tau_{l}$',
              r'$\tau_{s}$', r'$\tau_{c}$', r'$\tau_{\pi}$', r'$S_{pg}$',
```

```
              r'$P_{gg}$', r'Deadweight loss', r'$(1-\psi)r_{b}B$']
sankey.add(flows=flows2, labels=labelsCita, label='Public sector',
           orientations=[-1, -1, -1, -1, -1, -1, -1, -1, -1, -1, -1, -1,
                         -1, 1, 1],
           prior=0, connect=(0, 0))
plt.legend(loc='best')
plt.legend(fontsize=6)
diagr = sankey.finish()
```

The most important part of this snippet are the arguments of the second addition to the Sankey diagram *prior* and *connect*. We can read from the documentation:
prior(int): Index of the prior diagram to which this diagram should be connected.
connect(int, int): A (prior, this) tuple indexing the flow of the prior diagram and the flow of this diagram which should be connected. If this is the first diagram or prior is None, connect will be ignored. [5]

Next, we show the file **Figure4cpo**, that contains the equations presented in the Model subsection, and it is the file passed to **fsolve** to implement the Newton-Raphson method.

```
# function Figure4cpo(x0,param)
import numpy as np


def Figure4cpo(x0, param):
    # Parameter's binding
    alphap, alphag, alphal, deltap, deltag, thetaCgi, thetaL, thetaCpg, \
        thetaI, thetaLgp, mu, eta, tauc, taul, tauk, taus, taupi, sgg, \
        spg, L, A, ratGY, rb, gamma, Gamma = (param[i] for i in range(25))

    # Variable's binding
    Kpss, Kgss, Lpss, Lgss, Bss = (x0[i] for i in range(5))
    f = np.zeros(5)

    # Ecuations of the model
    Lgpss = thetaLgp*Lgss # (eq. 19)
    Lggss = Lgss-Lgpss # (eq. 18)
    Ipss = deltap*Kpss # (eq. 9)
    Igss = deltag*Kgss # (eq. 10)
    Lag = mu*Lpss**eta+(1-mu)*Lgpss**eta # (eq. 8 split)
    Kag = Kpss**alphap*Kgss**alphag # (eq. 8 split)
    Yss = A*Kag*Lag**(alphal/eta) # (eq. 8)
    Gss = ratGY*Yss # (eq. 21)
    rss = alphap*A*Kpss**(alphap-1)*Kgss**alphag*Lag**(alphal/eta) # (eq. 15)
    wss = (alphal*A*mu*Lpss**(eta-1)*Kag*Lag**(alphal/eta-1))/(1+taus) # (eq.16)
    Cgiss = thetaCgi*Gss # (eq. 3)
    Ygss = Gamma*Cgiss**gamma*Lggss**(1-gamma) # (eq. 11)
```

```
pggss = (Cgiss+(1+taus)*wss*Lggss)/Ygss # (eq. 13)
Cggss = pggss*Ygss # (eq. 12)
Cpgss = thetaCpg*Gss # (eq. 4)
Cpss = Yss-rb*Bss-Ipss-Igss-Cgiss-Cpgss # (eq. 22)
PIss = Yss-(1+taus)*wss*Lpss-rss*Kpss   # (eq. 14)
IFss = tauc*Cpss+(taul+taus)*wss*L+tauk*(rss-deltap)*Kpss+taupi*PIss + \
    (1-spg)*Cpgss+(pggss-sgg)*Cggss # (eq. 2)

# Newton's system
f[0] = Gss+rb*Bss-IFss # (eq. 1)
f[1] = Igss-thetaI*Gss # (eq. 5)
f[2] = Lpss+Lgss-L # (eq. 17)
f[3] = (rss-deltap)*(1-tauk)-rb # (eq. 20)
f[4] = Lgss*wss*(1+taus)-thetaL*Gss # (eq. 6)

return f
```

References

[1] de-Córdoba G.F., B. Molinari and J.L. Torres (2021), The Government in SNA-compliant DSGE Models. The B.E. Journal of Macroeconomics

[2] https://www.geeksforgeeks.org/pyplot-in-matplotlib/

[3] Quadrini V. and José-Víctor Ríos-Rull 2015. Section 14.2.1.1 The Irrelevance of Income and Wealth Inequality in the Neoclassical Model, in Handbook of Income Distribution. Elsevier

[4] https://numpy.org/doc/stable/user/absolute_beginners.html

[5] https://matplotlib.org/stable/api/sankey_api.html
